# Transverse susceptibility of nickel thin films with uniaxial anisotropy

**DOI:** 10.1038/s41598-021-82949-z

**Published:** 2021-02-04

**Authors:** Scott A. Mathews, Christopher Musi, Nicholas Charipar

**Affiliations:** 1 Materials Science and Technology Division, Naval Research Laboratory, Washington, DC 20375 USA; 2 NOVA Research Inc, 1900 Elkin Street, Alexandria, VA 22308 USA

**Keywords:** Materials science, Physics

## Abstract

A finite temperature Stoner–Wohlfarth model has been used to calculate the transverse susceptibility of an ensemble of ferromagnetic particles with distributed anisotropy. The simulated transverse susceptibility is in excellent agreement with data acquired from thin film samples of elemental nickel, deposited on 128° Y-cut LiNb0_3_. A strong, well-defined, uniaxial anisotropy is induced in the nickel film by low temperature annealing. Three peaks in the transverse susceptibility are observed in both the measured and simulated data when the applied field is misaligned with the hard axis by a few degrees. Two broad, reversible peaks occur when the applied field is equal to the anisotropy field. A single, sharp irreversible peak occurs when the absolute value of the applied field is less than the anisotropy field, and is associated with a metastable magnetic state. The irreversible peak disappears when the applied field is well aligned with the hard axis. The observed transverse susceptibility is consistent with the theoretical predictions of Aharoni et al*.* and is therefore consistent with the Stoner–Wohlfarth model.

## Introduction

Transverse susceptibility (TS) is a well-known method for directly determining the magnetic anisotropy in particulate magnetic systems^1^ and has been used to characterize a wide variety of magnetic recording materials^2^. The concept of TS was first introduced by Gans^3^ in 1909, and further developed by Aharoni et al*.*^4^ in 1957. The work of Aharoni, which was based on the coherent rotation model of Stoner and Wohlfarth^5^, predicted the appearance of peaks or cusps in the TS curves, occurring at the anisotropy and switching fields. These so-called Aharoni singularities eluded researchers until 1987, when Pareti and Turilli^6^ presented experimental verification of the phenomenon. Subsequently, in 1987, Le Dang et al*.*^7^ demonstrated that the peaks in the TS curves could be used to determine the magnitude and angular dispersion of the anisotropy field of thin films.

In this work, TS measurements are performed on nickel thin films, deposited on 128° Y-cut LiNb0_3_ substrates and annealed at low temperature. The films exhibit a strong uniaxial anisotropy^8^ and hysteresis branch crossing (HBC)^9^. The results are compared to numeric solutions to a finite temperature, Stoner–Wohlfarth (SW) model using a distribution of anisotropy magnitudes. While the SW-model was originally presented to model single domain particles, it is applicable to multi-domain particles, provided that the magnetization proceeds by coherent rotation and the motion of domain walls can be neglected^5^.

## Simulation

The simulations performed in this work assume that the magnetization is confined to the plane of the film by shape anisotropy. Therefore, only in-plane magnetic behavior is modeled. The SW-model is used to calculate the magnetic free energy (or energy landscape) as a function of the orientation of the magnetization based on two terms: the anisotropy energy and the Zeeman energy. The equilibrium orientation of the magnetization corresponds to a minimum of the energy landscape. The original SW-model assumes a zero-temperature approximation, meaning that if the magnetization lies in a local energy minimum, it will remain in the local minimum as the applied field is changed. When the applied field is changed such that the local energy minimum vanishes, the orientation of the magnetization will jump, irreversibly, to the global energy minimum. The finite temperature SW-model^9,10^ takes into account that when two minima exist in the energy landscape, the occupation probability of the magnetization (jumping from the local minimum to the global minimum and vice versa) is governed by Boltzmann statistics.

Once an algorithm calculating the numeric solution to the finite temperature SW-model has been implemented, this algorithm can be used to simulate a distributed anisotropy. The behavior of an ensemble of non-interacting particles with a distribution of anisotropy magnitudes or anisotropy directions can be modeled as the weighted sum of individual simulations.

Because the simulation allows the calculation of the equilibrium orientation of the magnetization (θ_eq_) and the curvature of the energy landscape (d^2^E/dθ^2^) in the vicinity of equilibrium, it allows the calculation of the transverse susceptibility, as shown below.

**Figure 1:**
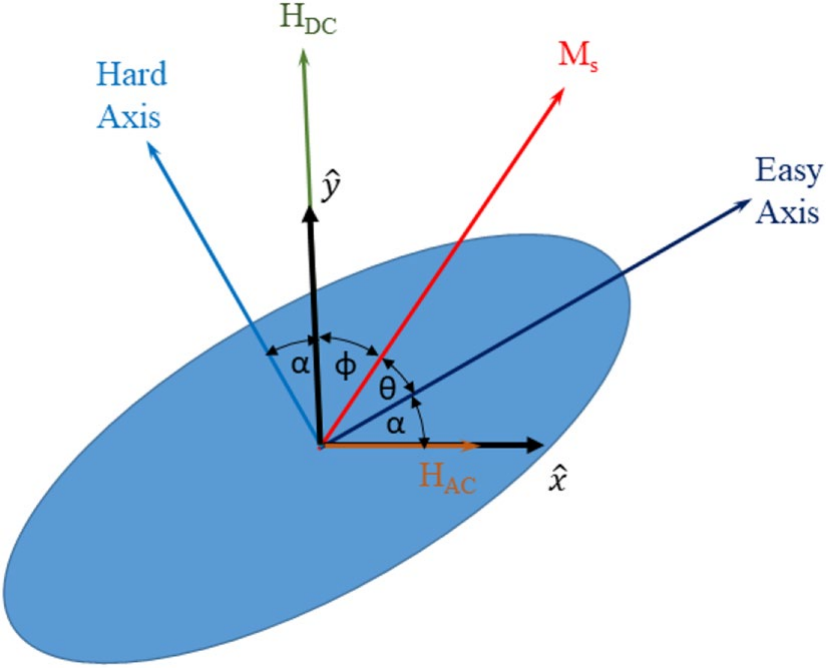
Geometry for the simulation of the SW-model and calculation of the transverse susceptibility.

Assuming a DC field along the y-direction and a small AC field (H_x_) along the x-direction, the transverse susceptibility is defined as

$$\chi_{trans} = \left. {\left( {\left| {\frac{{dM_{x} }}{{dH_{x} }}} \right|} \right)} \right|_{{H_{x} = 0}}$$

which can be written as

$$\chi_{trans} = \left| {\left( {\left. {\frac{{dM_{x} }}{d\theta }} \right|_{{\theta (H_{x} = 0)}} \cdot \left. {\frac{d\theta }{{dH_{x} }}} \right|_{{H_{x} = 0}} } \right)} \right|$$

or

1 $$\chi_{trans} = \left| {\left( {\left. {\frac{{dM_{x} }}{d\theta }} \right|_{{\theta = \theta_{eq} }} \cdot \left. {\frac{d\theta }{{dH_{x} }}} \right|_{{H_{x} = 0}} } \right)} \right|$$

where M_x_ is the component of the magnetization in the x-direction. From the geometry in Fig. 1 the value of M_x_ is

$$M_{x} = M_{s} \cos (\theta + \alpha )$$

and, taking the derivative of M_x_ with respect to θ and evaluating at θ = θ_eq_ yields

2 $$\left. {\frac{{dM_{x} }}{d\theta }} \right|_{{\theta = \theta_{eq} }} = - M_{s} \sin \left( {\theta_{eq} + \alpha } \right)$$

Using the first three terms of a Taylor expansion of the magnetic energy as a function of θ in the vicinity of equilibrium (θ_eq_) yields

3 $$E\left( \theta \right) \cong E\left( {\theta_{eq} } \right) + \left. {\frac{dE}{{d\theta }}} \right|_{{\theta_{eq} }} \left( {\theta - \theta_{eq} } \right) + \left. {\frac{1}{2}\frac{{d^{2} E}}{{d\theta^{2} }}} \right|_{{\theta_{eq} }} \left( {\theta - \theta_{eq} } \right)^{2}$$

At equilibrium, the first derivative of energy with respect to θ is zero. Taking the derivative of Eq. (3) with respect to θ yields

4 $$\frac{dE}{{d\theta }} = \left. {\frac{{d^{2} E}}{{d\theta^{2} }}} \right|_{{\theta_{eq} }} \left( {\theta - \theta_{eq} } \right)$$

The torque on the magnetization at equilibrium due to H_x_ is

$$\tau = \overrightarrow {{M_{s} }} \times \overrightarrow {{H_{x} }} = M_{s} H_{x} \sin \left( {\theta_{eq} + \alpha } \right)$$

By definition, the torque is equal to the derivative of energy with respect to angle

5 $$\frac{dE}{{d\theta }} = \tau = M_{s} H_{x} \sin \left( {\theta_{eq} + \alpha } \right)$$

Substituting Eq. (5) into Eq. (4) yields

6 $$\left. {\frac{{d^{2} E}}{{d\theta^{2} }}} \right|_{{\theta_{eq} }} \left( {\theta - \theta_{eq} } \right) = M_{s} H_{x} \sin \left( {\theta_{eq} + \alpha } \right)$$

Rearranging Eq. (6) yields

7 $$\theta = \frac{{M_{s} H_{x} \sin \left( {\theta_{eq} + \alpha } \right)}}{{\left. {\left( {\frac{{d^{2} E}}{{d\theta^{2} }}} \right)} \right|_{{\theta_{eq} }} }} + \theta_{eq}$$

Taking the derivative of Eq. (7) with respect to H_x_ and evaluating at H_x_ = 0 yields

8 $$\left. {\frac{d\theta }{{dH_{x} }}} \right|_{{H_{x} = 0}} = \frac{{M_{s} \sin \left( {\theta_{eq} + \alpha } \right)}}{{\left. {\left( {\frac{{d^{2} E}}{{d\theta^{2} }}} \right)} \right|_{{\theta_{eq} }} }}$$

Substituting Eqs. (8) and (2) into Eq. (1) yields an expression for the transverse susceptibility:

9 $$\chi_{trans} = \left| {\left( {\left. {\frac{{dM_{x} }}{d\theta }} \right|_{{\theta = \theta_{eq} }} \cdot \left. {\frac{d\theta }{{dH_{x} }}} \right|_{{H_{x} = 0}} } \right)} \right| = \left| {\frac{{M_{s}^{2} \sin^{2} \left( {\theta_{eq} + \alpha } \right)}}{{\left. {\left( {\frac{{d^{2} E}}{{d\theta^{2} }}} \right)} \right|_{{\theta_{eq} }} }}} \right|$$

The TS was simulated using a finite temperature, SW-model with a distributed anisotropy magnitude. The details of the simulations are given in^9^. For each value of applied field, the simulation computes the energy landscape (energy as a function of in-plane orientation of the magnetization), the equilibrium orientation of the magnetization, and the curvature of the energy landscape at the equilibrium orientation. The TS is computed from Eq. (9). When two energy minima exist in the energy landscape, the occupations are determined using Boltzmann statistics, assuming a temperature of 300 K. The total TS is computed as the weighted sum of the TS values of the two energy minima. The simulation assumes an ensemble of Stoner–Wohlfarth particles having a Gaussian (normal) distribution of anisotropy fields with a standard deviation of (σ_Hk_). The TS is simulated over a range of anisotropy fields and the TS of the ensemble is calculated as the weighted sum over the distribution. The free parameters of the simulation are: the average anisotropy field (H_kAve_), the standard deviation of the anisotropy field (σ_Hk_), and the effective particle volume (V_eff_). The values of H_kAve_ = 520 Oe, σ_Hk_ = 20 Oe, and an effective particle volume of V_eff_ = 7.5 × 10^–16^ cm^3^ are found by adjusting these parameters to fit the data, and are consistent with other measurements^8,9^.

## Experimental

Nickel films were deposited by DC magnetron sputtering on polished 128° Y-cut LiNbO_3_ substrates. The nickel layers were between 90 and 100 nm thick, as measured by stylus profilometery. Samples were thermally annealed under vacuum (< 10^–6^ Torr) at 325 °C for 2 h. The nickel films were patterned into 7 mm diameter circles using conventional photolithography and wet-etch, in order to remove any in-plane shape anisotropy.

In-plane TS measurements were performed at room temperature in a custom built vibrating sample transverse susceptibility (VSTS) system, employing a 1.5 MHz, LC-oscillator and an electromagnet with automated control. The LC-oscillator used a rectangular solenoid coil (5 mm × 25 mm × 25 mm) of approximately 60 turns and associated electronics. The frequency of the LC-oscillator was measured using a commercial frequency counter. The LC-oscillator was fixed to the electromagnet between the pole pieces, with the axis of the rectangular solenoid perpendicular to the field of the electromagnet, such that the electromagnet and LC-oscillator rotated as a single unit. Samples were mounted on the end of a wooden dowel attached to a reciprocating mechanism that moved the samples into and out of the rectangular solenoid coil at 15–17 Hz. The TS signal was assumed to be proportional to the change in frequency of the LC-oscillator (Δf = f_out_ − f_in_)^6^. The TS signal was acquired using a “step-and-measure” technique with a field step of approximately 50 Oe. The sample and reciprocating mechanism were fixed in orientation, while the electromagnet and LC-oscillator were rotated in 1° steps in the vicinity of the hard axis.

## Results

**Figures 2:**
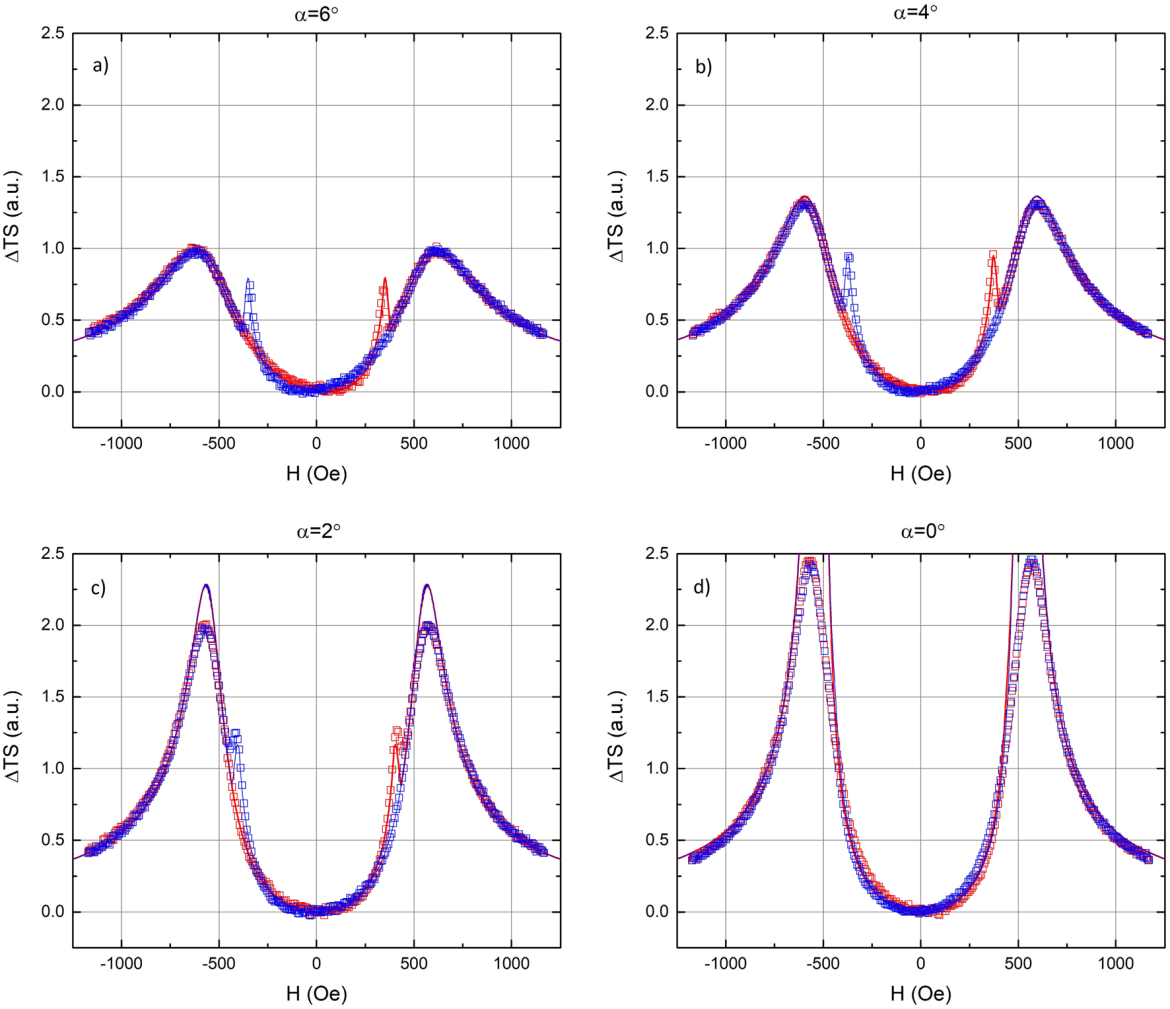
(**a**–**d**) Change in transverse susceptibility (with respect to zero field) vs. applied DC field for four orientations. Ascending and descending curves are shown in red and blue, respectively. Open squares represent measured data, while solid lines represent the simulation.

Figures 2a–d show the calculated and measured change in TS as a function of applied field for four misalignment angles (α = 6°, 4°, 2°, 0°). As shown in Fig. 1, the misalignment angle (α) is defined as the angle between the hard axis and the applied DC field (or, equivalently, the angle between the easy axis and the AC field). Data for increasing and decreasing field are shown in red and blue, respectively. Both the calculated and measured data are normalized to the maximum TS value at α = 6°.

## Disscusion

Both the measured ΔTS and the calculated ΔTS show three peaks when the applied field is close to, but not on the hard axis and is swept from positive to negative (or negative to positive) saturation. The two peaks at approximately ± 520 Oe are reversible while the sharper peak at approximately ± 350 Oe is irreversible. When the applied field is aligned with the hard axis, only the two reversible peaks remain (Fig. 2d.). This is the exact behavior predicted by Aharoni et al*.*^4^ for a single, SW-particle. As shown by Aharoni et al*.*, the two reversible peaks in the TS are due to the flattening out of the energy landscape when the component of the applied field in the direction of the hard axis exactly cancels the anisotropy field. When the applied field is aligned with the hard axis, the SW-model predicts that the TS becomes infinite; the so-called Aharoni singularity. Obviously, the measured TS always remains finite. This is due to the fact that the theory assumes an infinitesimally small transverse AC field, whereas the AC field in a real measurement is finite. The finite value of the transverse AC field accounts for the discrepancy in peak heights shown in Fig. 2c,d.

**Figure 3:**
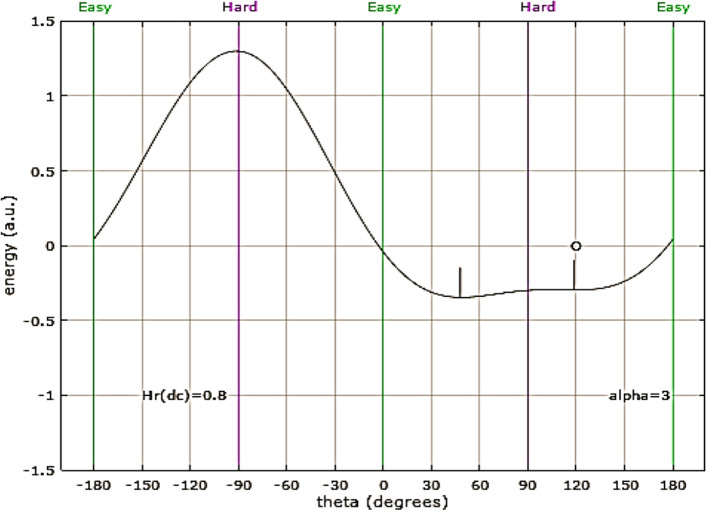
Energy as a function of orientation of magnetization (energy landscape) for an SW-particle with a field of 0.8H_k_ applied 3° from the hard axis. The short, vertical lines show the locations of the minima, while the letter “o” shows the orientation of the magnetization at T = 0 K.

**Figure 4:**
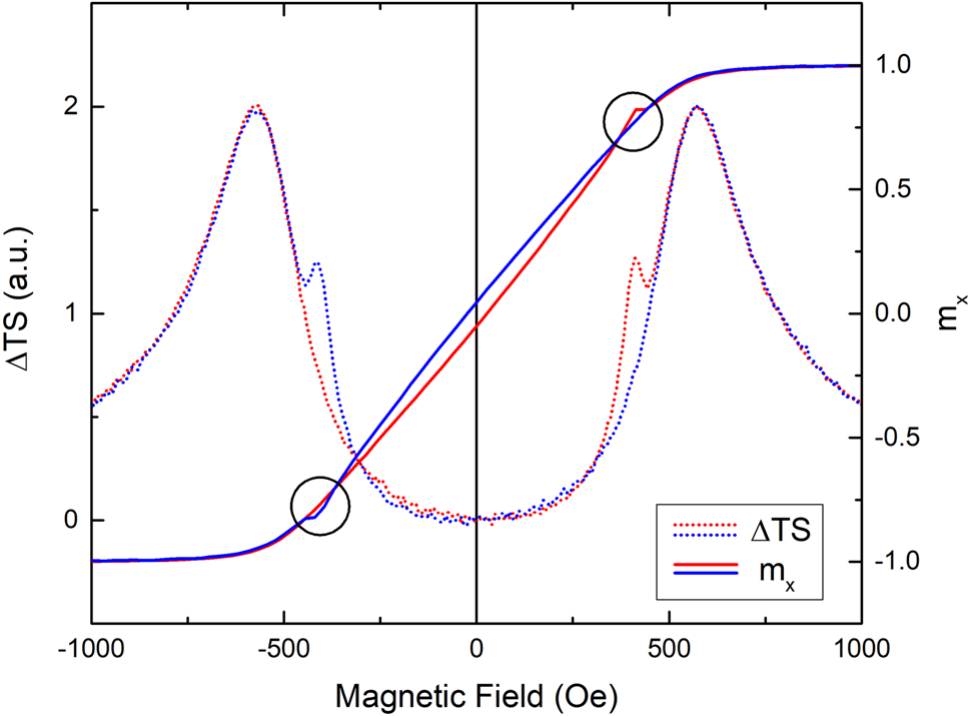
The reduced x-component of the magnetization (m_x_ = M_x_/M_s_) and the change in TS as a function of applied field 2° from the hard axis. Ascending and descending curves are shown in red and blue, respectively. Black circles highlight the hysteresis branch crossings. Note that the crossing of hysteresis branches occurs at the same applied field as the irreversible TS peak.

The irreversible peak, visible only when the applied field is slightly misaligned with the hard axis, is associated with the flattening out of the energy landscape around a metastable, local energy minimum. As the field is increased, this local minimum flattens out, and then disappears, resulting in an irreversible change in the magnetization. This flattening out of the local minimum is responsible for the irreversible peak in the TS and the hysteresis branch crossing^9^ reported elsewhere. This situation is depicted graphically in Fig. 3, which shows the energy as a function of the orientation of the magnetization. In Fig. 3, a field of 0.8H_k_ is applied 3° from the hard axis. The two short, vertical lines show the locations of the two energy minima. The letter “o” at approximately 120° indicates the “occupation”; the orientation of the magnetization at 0 K. Figure 3 represents the magnetic configuration just before the magnetization jumps irreversibly from the local minimum to the global minimum. The occupied local energy minimum has flattened out, leading to a large increase in TS and the crossing of hysteresis branches. Figure 4, which shows both the x-component of the reduced magnetization and the change in TS, demonstrates the crossing of hysteresis branches with the field 2° from the hard axis. Figure 4 shows that the irreversible peak in TS and the crossing of hysteresis branches occur at the same applied field. The irreversible peak in the TS is a necessary, but not sufficient condition for the crossing of hysteresis branches^9^, as the irreversible TS peak is shown to persist at misalignment angles where the branch crossing has vanished.

## Conclusions

We have shown that the temperature dependent Stoner–Wohlfarth model accurately predicts the transverse susceptibility of nickel films annealed on lithium niobate substrates. In addition, we have shown that the measured transverse susceptibility agrees with the theoretical results of Aharoni et al*.*, and that the two reversible peaks provide a direct measurement of the anisotropy in the films. Our modelling indicates that the same underlying physics, the flattening out of the local energy minimum, is responsible for both the irreversible peak in the transverse susceptibility and the hysteresis branch crossing (reported elsewhere).
